# The Association Between Health Conditions, Consciousness, Involvement, and Knowledge and Dietary Supplement Intake among University Students in South Korea

**DOI:** 10.3390/ijerph16204028

**Published:** 2019-10-21

**Authors:** Jinkyung Choi

**Affiliations:** Department of Foodservice Management, Woosong University, Daejeon 34606, Korea; choi3728@wsu.ac.kr; Tel.: +82-42-630-9453

**Keywords:** dietary supplements, health involvement, consciousness, health condition, health behaviors, knowledge

## Abstract

Dietary supplements (DSs) are typically used by the elderly in a population, but younger age groups are increasingly purchasing these products. In consideration of this issue, the present study investigated the DS-related behaviors and general lifestyles of university students in South Korea. The health conditions, consciousness, involvement, and knowledge, as well as the future behavioral intentions, of DS users and non-users were determined and compared. A survey was administered to the respondents, and measurements were adapted and rephrased to suit the Korean context. Results showed that although the behaviors of DS users and non-users were characterized by similar patterns, significant differences in health involvement and future purchase were found between these groups. Health involvement influenced DSs buying intentions in the future, but no significant differences in the other variables were found. The findings suggested that university students take DSs regardless of their expectations about their efficacy and that their knowledge does not affect their intention to purchase such products.

## 1. Introduction

Increased life expectancy encourages individuals to lead healthy lives, or perhaps healthy lifestyles allow individuals to have extended life expectancy. People have shown interest in how to pursue a wholesome manner of living and accordingly engage in exercise and consume healthful food, including dietary supplements (DSs) [1]. These products are used by an average of 50% of adolescent populations in developed countries, with consumption in the US and the UK being especially high [2]. Approximately 54% of American adults use DSs such as multivitamins and minerals [3].

In the Korean context, about 42% of adults have taken a DS at least once in their lifetime, as indicated in the results of the Korean National Health and Nutrition Examination Survey in 2015 (KNHANES) [4]. The market for DSs has rapidly increased since 2009, reaching a size of US$1792 million in 2013 [5]. Vitamin or mineral products are the most frequently consumed supplements in the country [6]. In 2015, the total healthy eating index (HEI) score for Koreans was 57 points [7]. Similar to the Korean HEI score, the total HEI score for Americans was 53.5 points between 2007–2008 [7]. The Korean HEI indicated that although Koreans had a high intake of vegetables, protein, and carbohydrates, their diet was low in sodium and whole grains. Moreover, according to the 2017 Korean National Health and Nutrition Examination Survey (KNHANES), the 19–29 age group had the highest percentage of malnutrition compared with other age groups [8]. From 2016–2017, Korean people’s intake of fat, sodium, vitamin A, and vitamin C decreased by more than 5% [8].

DSs are used by many adolescents [9], such as college students, who are transitioning between adolescence and adult life and still have dietary habits from high school. Such usage is prompted by adjustments to university life, in which behaviors and decisions are less regulated than those in high school life. This transition to university life may therefore result in important changes in dietary choices [10] and dietary patterns [11,12]. Dietary patterns have been strongly associated with DS use [11,13], which is compelled most strongly among college students by the desire to promote general health and increase energy [13]. Patterns of DS consumption showed that about two-thirds of surveyed college students regularly use these products in U.S. [13]. College students in particular take DSs in order to relieve stress and enhance their health as a result of self-decision making and unbalanced dietary habits [5]. However, dependency on DSs may build unhelpful dietary habits for the rest of their lives [5]. In Korea, 36.1% of individuals aged 19 to 29 years consume DSs—a proportion less than that accounted for by other age groups but more than that of people aged 10 to 18 years in 2015 [4]. DSs user adults responded 27.2% for men and 40.2% of women take DS for longer than two weeks during a year [14]. DSs are recommended to consume on a daily basis however this recommendation has not been received well by consumers in somehow. Note, however, that among these groups, college students engage in healthy behaviors such as physical activities, nutritious diet, quality sleep, or stress management to a lesser extent [15]. Once established, lifetime dietary habits may strongly influence the rest of the individual’s dietary style. Individuals in this age group develop and build their own food choices. To a lesser extent, family involvement and the surrounding environment affect their dietary habits, which are in the finalizing steps in the process of building up whole lifetime dietary habits. Increased fast food consumption and eating out causes chronic diseases. 

Behaviors related to DS intake differ from those associated with the consumption of food. Typically, food is considered a low-involvement product [16], whereas DSs require high involvement given the accompanying extra health benefits that go beyond those offered by food options [17]. Involvement refers to the degree of interest and consideration that an individual devotes to a subject [18]. Involvement behaviors are related to health or dietary options; specifically, one’s consideration of health issues, such as variety in healthy meals, nutritional value, and meals that support personal dietary plans, is relevant to the decision to opt for healthy dining [19]. The additional health advantages presented by DS consumption should be attributed in part to the involvement that such usage entails [17]. This argument is supported by a study wherein the level of consumer involvement in the purchase of organic food products was found related to the intention of the customers to purchase such commodities [20].

Individuals become more health conscious as they are increasingly concerned about their well-being and quality of life. Research suggested that health-conscious individuals tend to more strongly espouse a prevention-oriented attitude than do those who are less health conscious. A study that measured the impact of health consciousness on consumer attitudes toward DSs found that these products are effective [21]. Health consciousness factors in proactive behaviors to improve or maintain health [22]. Individuals modify the manner by which they conduct themselves with help from positive stimuli or motivation; negative stimuli may cause behaviors that deviate from healthy choices and thus impede favorable outcomes [23]. Health consciousness may thus be a key determinant of DS consumption and the search for related information, which is abundantly available from a variety of media sources; the problem is that such information is often contradictory and confusing [13].

The judgment of whether health information exerts positive or negative effects requires knowledge. Knowledge regarding healthy ways to eat, for example, is essential in choosing what to consume [24,25]. Nevertheless, the effects of knowledge on food choices are a controversial issue [12,26]. In the case of college students, for instance, although they understand that consuming fast food can cause diseases, their knowledge about such matter is not a factor that influences their consumption choices [12,27]. The food-related behaviors of these young adults are considerably affected primarily by taste, convenience, and cost, with healthiness being last on this list [28,29,30]. Students spend a substantial amount of time on campus, where the environment that surrounds sustenance, such as available food, in terms of variety, price, and convenience may tremendously affect how they behave with respect to food consumption. On the basis of the discussions above, this study investigated the DS-related behaviors and general lifestyles of university students in South Korea. To this end, the health conditions, consciousness, involvement, and knowledge, as well as the future behavioral intentions, of DS users and non-users were ascertained and compared.

## 2. Materials and Methods

### 2.1. Participants

This analytical cross-sectional study recruited university students in South Korea, excluding those who were undergoing medical treatment for health problems. Before the survey, the study questionnaire was approved by the institutional review board (1041549-190709-SB-77) at Woosong University. After the selected respondents expressed agreement to participate in the research, a survey was administered to them. A total of 310 questionnaires were distributed, and 289 were returned. After careful screening, four questionnaires were further eliminated because these were completed by students undergoing medical treatment. Responses which most questions were answered by respondents were entered into a final sample to analysis. A final sample of 285 questionnaires were obtained for analyses. On DS experience, 179 respondents (63.5%) had experience, while 103 (36.5%) had not taken DSs previously. Of the DS-experienced respondents, 89 (49.7%) were taking DSs currently and 90 (50.3%) were not taking DSs.

The sample size appropriate for this study was determined via the G*Power program [31], which uses a *p* < 0.05, a power of 95%, an effect size of 0.15, and four independent variables as components of multiple regression analysis. The calculated size was 129 respondents. The conducted two-tailed *t*-test indicated that a sample size of 54 participants is required on the basis of an effect size of 0.5 and a power of 95, whereas chi-square statistics estimated this requirement at 220 respondents. The consideration of a 10% dropout rate yielded a required sample of 242 participants, which was the size adopted in this study.

### 2.2. Measurements

A self-administered questionnaire was used in data collection, which commenced in July 2019. The instrument contains DSs usage information, general health conditions, perceptions towards health behaviors, nutritional knowledge, and demographics. Questions asking DSs usage were excerpted from [32]. The questionnaire also consists of four health-related statements to be rated on a five-point Likert scale (1: *not very good*, 5: *very good*), which were also adapted from previous research [32,33]. The four questions that focus on health conditions were based on [17], and an additional four queries into health consciousness were obtained from [17] and Involvement seems to be dynamic, exhibiting characteristics that are specific to the issue of interest in this study. Hence, this research measured involvement on the basis of health information search behaviors. Four questions on this matter were taken from [17]. Questions on future purchase intent was adapted from [32]. All the questions were asked to rate some of the statements on a five-point Likert scale (1: *strongly disagree*, 5: *strongly agree*). 

To measure individual knowledge, five questions were excerpted from previous studies [34]. Knowledge was measured using five questions, with a correct answer coded as 1 and an incorrect response coded as 0. The correct responses were added up per respondent then divided by the number of questions, which was 5. Therefore, the mean values were calculated.

Body mass index was calculated on the basis of self-reported height (cm) and weight (kg) and were categorized as follows: below 18.5 = low weight, 18.5 to 22.9 = normal weight, 23 to 24.9 = overweight, and 25 = obese.

The respondents were asked questions regarding DS consumption. Responses of “yes” and “no” to the question of whether one has ever taken DSs were coded as 1 and 0, respectively. The respondents with DS consumption experience were queried as to (1) whether they were currently taking DSs, (2) whether they were experiencing any side effects, (3) what their preferences are in purchasing DSs, (4) what products they have consumed, and (5) what their reasons are for taking DSs. The demographic characteristics of the participants were also obtained.

### 2.3. Statistical Analyses

The data were analyzed using the Statistical Package for the Social Sciences version 25.0 (IBM, Chicago, IL, USA). Descriptive statistics were used to illustrate the respondents’ demographic characteristics and experience with DSs. Cronbach’s alpha analysis was run on three independent variables (health condition, consciousness, involvement) to test the validity of the constructs. Independent *t*-tests were performed to ascertain the differences in health-related behaviors between DS users and non-users. A multiple regression was conducted to probe into the effects of health-related behaviors and knowledge on DS purchase.

## 3. Results

### 3.1. Demographics

The demographic characteristics of the respondents are shown in Table 1. More than half of the respondents were female (57.5%) and single (98%). The sample was composed mostly of sophomores (40.8%), followed by freshmen (30.3%), juniors (17.3%), and seniors (11.6%). The majority of the respondents were aged 20 (28.4%) and 21 (28.8%) years. About 54% lived on their own, and 37% resided with their parents. Out of the participants, approximately 30% were on a diet; most of them (41.2%) had neutral opinions regarding their body image, whereas 38.7% expressed dissatisfaction with such attribute. Only 7% were satisfied with how their bodies look. The majority of the respondents (61.2%) were of normal weight, and 16.4% were overweight. 

College majors, the respondents were divided into those pursuing food- and health-related programs and those studying under non-food and non-health disciplines. All the correct answers of each respondent were summed (*n* = 275, M = 0.83, SD = 0.79). The *t*-tests showed no significant differences in level of knowledge between the food/health and non-food/non-health majors (*t* = 1.416, df = 273, *p* > 0.05).

### 3.2. DSs Intake Behaviours

Table 2 presents the results of the descriptive analyses of experience with DS. More than half of the respondents (63.5%) have taken DSs in their lifetime, whereas 36.5% have never consumed such products. Of those who have taken DSs, almost half (49.7%) were taking DSs at the time the study was being conducted. The students stated that they were taking DSs because these were recommended by their families (45.1%) and because they wanted to promote general health (34.7%). Some of the respondents had no reason driving their consumption (5.8%). 

The participants typically checked nutritional information on packaging (48%) and acquired information from others (28.7%) when deciding on purchasing DSs. About 88% have not experienced side effects when taking DSs, and only a small number of them encountered problems such as digestion issues (5.5%), fast heart beat (2.5%), constipation (2.5%), and headache (1.2%). Multiple responses were derived when the participants were asked about the specific DSs that they have taken. The most commonly consumed supplements were multivitamins (30.3%), followed by vitamin C (17.3%), probiotics (15%), omega-3 products (11.1%), eye-care supplements such as lutein (6.5%), iron (5.5%), protein supplements (5.5%), supplements that support diet (4.9%), and calcium (3.9%).

### 3.3. Assessment of Health Conditions, Involvement and Knowledge

Four constructs, namely health conditions, health consciousness, health involvement, and future behavioral intention were used to investigate consumers’ perception and behavior towards DSs. Three constructs, namely, health conditions (Cronbach’s alpha = 0.839), health consciousness (Cronbach’s alpha = 0.675), and health involvement (Cronbach’s alpha = 0.875), satisfied the validity criteria for measurement. Generally, the respondents exhibited a neutral stance as regards their health conditions (M = 3.16, SD = 0.864), physical strength (M = 2.90, SD = 0.896), psychological conditions (M = 3.20, SD = 0.838), and ability to take care of themselves (M = 3.35, SD = 0.861). Similar patterns were found for health consciousness.

The health conditions, health consciousness, health involvement, and future behaviors of the DS users and non-users were compared using independent *t*-tests (Table 3). Each of the constructs exhibited high validity as indices, and the results showed significant differences between the groups in terms of health involvement (*p* < 0.001) and future behavioral intention (i.e., purchase) (*p* < 0.001). However, no such dissimilarities were found with respect to health conditions and health consciousness. The DS users engaged more frequently in information searches than did the non-users, and the former also exhibited an intention to purchase DSs in the future.

The correlations among the four constructs are shown in Table 4. A multiple regression was run to ascertain which health-related behaviors affected the intention to purchase DSs (F = 46.456, Adj. R^2^ = 0.40, R^2^ = 0.406, *p* < 0.001). The findings showed that health involvement highly affected future DS purchase (B = 0.756, SE = 0.629, *t* = 12.721, *p* < 0.001), whereas health conditions, health consciousness, and knowledge exerted no effects (Table 5).

All questions asked on 5-point Likert scale (1: very disagreed, 5: very agreed).

## 4. Discussion

This study found that more than half of respondents have taken DSs, and out of these groups, almost half were taking the aforementioned supplements during the course of the research. This result is similar to the outcome of the KNHANES, which indicated that about 42% of Korean adults have taken a DS at least once in their life [5]. Interestingly, the most important driver of DS consumption is recommendation from family members. College students’ behaviors, such as valuing their peers’ opinions, were in line with adolescent behaviors. A previous study found that most college students who took DSs were usually recommended to by family and friends [32]. This study also found similar outcomes. University students are still in the transition stage between adolescence and adulthood and may therefore continue to need extra care from their families. Adolescents are a most vulnerable and easily misinformed population [9], who are targeted by the DS industry [35]. Suggestions from friends also considerably influence the health behaviors of university students [32], who tend to emulate their peers. DS consumption is likewise prompted by the desire to promote general health. The transition from high school to college may result in alterations to dietary behaviors because of dramatic changes in surrounding environments [36,37,38]. To compensate for lifestyle adaptations, college students may consume DSs to maintain overall well-being [5].

The current work found that nutritional information on product packaging was the most important factor for the consideration of DS purchase. Consumers search for information when they make decisions [39,40] because of the many choices and products that are available to them [41]. An intriguing finding in this research is that although the respondents’ knowledge levels were low, they still carefully examined nutritional information on packaging, suggesting that some information can be misleading or some other reasons for consumers. Abundant information on DS products may confuse consumers, especially those who have low knowledge of these commodities. Many previous studies confirmed that knowledge level and health behaviors are irrelevant to consumers’ decision to purchase products [42]; that is, purchase is based on perceptions rather than objective evidence [1,43]. Hence, education that cultivates adequate knowledge about DSs is needed [43].

Recommendations from others accounted for the second most influential factor for DS consumption for the same reasons discussed in regard to family and peer influence. The most commonly consumed DSs were multivitamins, consistent with the findings of another study [6]. Meanwhile DS users and non-users significantly differed in terms of DS intake, but no such difference was found in connection with other health-related behaviors.

DSs can be beneficial to one’s health; if an individual is driven to take these products out of concern for his/her well-being, then such behavior may motivate other healthful habits, such as regular exercise. However, no differences were found between DS users and non-users. The relationship between stress and food intake is a well-known phenomenon. Food intake increases and shifts toward unhealthy choices when people are exposed to stressful conditions [44]. Concerns have been raised regarding the possibility that DSs will encourage individuals to eat less healthy food or pursue unhealthy lifestyles [13]. DS users may rely solely on supplements and neglect other health behaviors that may bring about positive health outcomes.

The prevalence of DS consumption has been attributed to health conditions and health consciousness. This study postulated that health consciousness significantly affects DS consumption, but general health conditions and health consciousness were viewed in a neutral manner by both the DS users and non-users. These individuals were highly confident about their health and physical strength. Given that DSs are dispensed without the need for a prescription and are available to all individuals who may have different health conditions, the benefits of such products may be minimal, and their efficacy remains controversial [6]. Therefore, although the DS users may have been aware of the potentially weak efficacy of DSs, they also held positive perceptions of the benefits of consuming these supplements. This positivity might have stemmed from advertisements that deliver highly persuasive messages in a fiercely competitive DS market [43]. The result can also be attributed to the DS users understanding that supplement consumption helps them keep pace with the demands of campus life [12], which is known to present easy access to rapidly available snacks, regardless of the health condition and consciousness of students.

Given that involvement is difficult to measure [17], information search behaviors were used instead. These behaviors were significantly more prevalent among the DS users than non-users. Involvement is related to certain manners of conduct because DS purchase requires considerable cognitive effort [17], in contrast to food purchase [17,20]. As previously mentioned, the DS users and non-users exhibited no difference in terms of health conditions and consciousness. Thus, individuals in need of DSs look for information and are finally led to purchase these supplements.

## 5. Conclusions

This study investigated DS usage behaviors among university students in South Korea. Abundant studies have examined the manner by which university students conduct themselves in connection with their diets, but few have been directed specifically toward shedding light on the effects of health conditions, consciousness, and involvement on DS intake among South Korean students. The present study found that surveyed college students believed that they were healthy [9] and only moderately conscious of their general health. Moreover, their health consciousness was unrelated to their information searching behaviors regarding their health. This may be the result of their lack of nutritional knowledge or due to strong social bonds with their peer group. Additionally, the results indicated that their DS consumption was highly influenced by recommendations from family and friends. Since they were not knowledgeable of nutrition, they would review the package’s nutritional information when deciding whether to make a purchase—this behavior suggests that they may purchase DSs that they do not need. Furthermore, certain statements on the packages may motivate and persuade them to make unnecessary purchases. Hence, to avoid reckless DS intake, proper educational interventions are necessary.

For the DS industry, fierce marketing is necessary for them to survive in the market. However, persuasive but vague statements should be prevented for these products to be effectively used in the next stage of university life for DS users. These students will enter the workplace and might need DSs more than ever for their health as they grow older. Achieving longevity in the DS market necessitates the formulation of policies regarding supplements.

The limitations of this research should be noted for future studies. First, this study was conducted in a campus in South Korea; thus, different university students around the world should be involved in future research. Second, campus lifestyles should also be investigated to examine the DS consumption and dietary habits of students. Third, to assess which areas require nutritional education, actual nutritional intake and the consumption of DSs should be measured. Finally, the specific ingredients of DSs and related behaviors should be examined to draw implications for the industry and the well-being of consumers taking DSs. The results of the present research may add to the DS literature as university students take these products, regardless of their health conditions or consciousness. Moreover, their knowledge levels do not affect their intention to buy DSs, thus raising the risk of misuse among the population. Appropriate educational intervention is necessary to enable university students to avoid adverse effects from DSs.

## Figures and Tables

**Table 1:** Characteristics of Demographics of the Respondents (*n* = 285).

Characteristics		Frequency (Valid Percentage)	DS-Users (Valid Percentage)	Non-Ds User (Valid Percentage)
Gender	Male	120 (42.5)	75 (62.5)	45 (37.5)
	Female	162 (57.5)	104 (64.2)	58 (35.8)
Marital status	Married	5 (2)	5 (100)	0 (0)
	Single	275 (98)	173 (62.9)	102 (37.1)
	Missing	2		
Grade	Freshman	86 (30.3)	56 (65.1)	30 (34.9)
	Sophomore	116 (40.8)	67 (59.3)	46 (40.7)
	Junior	49 (17.3)	35 (71.4)	14 (28.6)
	Senior	33 (11.6)	20 (60.6)	13 (39.4)
	Missing	1		
Place of residence	Live in board and lodging	4 (1.4)	4 (100)	0 (0)
	Live alone	154 (54.2)	98 (64.5)	54 (35.5)
	School dormitory	21 (7.4)	13 (61.9)	8 (38.1)
	Live with parents	105 (37.0)	64 (61.5)	40 (38.5)
	Missing	1		
Major	Food or health related	181 (63.5)	119 (66.1)	61 (33.9)
	Non-food or non-health related	104 (36.5)	60 (58.8)	42 (41.2)
Are you on a diet?	Yes	83 (29.2)	59 (71.1)	24 (28.9)
	No	201 (70.8)	120 (60.6)	78 (39.4)
	Missing	1		
Are you satisfied with your body figure?	Not very satisfied	33 (11.6)	20 (60.6)	13 (39.4)
	Not satisfied	110 (38.7)	74 (67.9)	35 (32.1)
	So-so	117 (41.2)	73 (63.5)	42 (36.5)
	Satisfied	20 (7.0)	11 (55.0)	9 (45.0)
	Very satisfied	4 (1.4)	1 (25.0)	3 (75.0)
	Missing	1		
	Below18.5	21 (8.4)	13 (61.0)	8 (38.1)
BMI	18.5~22.9	153 (61.2)	93 (61.6)	58 (38.4)
	23~24.9	41 (16.4)	26 (63.4)	15 (36.6)
	25 and above	35 (14.0)	23 (65.7)	12 (34.3)
	Missing	35		

**Table 2:** General Dietary Supplement Consumption Behaviors.

Questions	Answers	Frequency	Valid Percentage (%)
Have you ever taken dietary supplements	Yes	179	63.5
	No	103	36.5
	Missing	3	
Are you taking dietary supplements	Yes	89	49.7
	No	90	50.3
	Missing	106	
* Reason for taking dietary supplements	Promote general health	60	34.7
	No reasons	10	5.8
	Recommendations from medical experts for general health	3	1.7
	Recommendations from family	78	45.1
	Because of specific nutritional health promotion	20	11.6
	Because Friends taking dietary supplements	1	0.6
	Others	1	0.6
	Missing	6	
* Considerations when select dietary supplements	Price	8	4.7
	Brand	24	14
	Nutritional information on package	82	48
	Quantity	5	2.9
	Recommendations from others	49	28.7
	Advertisement	3	1.8
	Missing	8	
	Problems with digestions	9	5.5
	Hives	0	0
	Fast heart beating	4	2.5
* Symptoms of side-effect	headache	2	1.2
	constipation	4	2.5
	Fallen hair	1	0.6
	Kidney problems	0	0
	Inappetence	0	0
	others	0	0
	No experience with side effect	143	87.7
	Missing	16	

* Questions adopted from Sung and Choi [32].

**Table 3:** Comparisons of DS users and non-users of health conditions, consciousness, involvement, and future behavioral intention toward DS.

Specification of Features	Mean ± SD	DS Users (*n* = 179)	Non-DS User (*n* = 103)	*t*-Value
**Health conditions** (Cronbach’s alpha = 0.839)				
General health condition	3.16 ± 0.864	3.13 ± 0.844	3.19 ± 0.886	−0.565
General physical strength	2.90 ± 0.896	2.91 + 0.891	2.86 + 0.886	0.372
Psychological health condition	3.20 ± 0.838	3.18 + 0.822	3.22 + 0.851	−0.432
Ability to take care of yourself	3.35 ± 0.861	3.35 + 0.816	3.34 + 0.924	0.062
**Health consciousness** (Cronbach’s alpha = 0.675)				
Try to prevent health problems before I feel any symptoms	3.23 ± 0.878	3.27 + 0.826	3.17 + 0.971	0.997
Concerned about health hazards and try to prevent problems	3.31 ± 0.857	3.37 + 0.828	3.18 + 0.905	1.792
Not worried health problems unless I have any problems ^a^	2.94 ± 0.850	3.02 + 0.804	3.13 + 0.925	−1.041
Do not take any actions before I have health problems ^a^	3.00 ± 0.866	2.98 + 0.841	3.06 + 0.916	−0.750
**Involvement** (Cronbach’s alpha = 0.875)				
Pay attention to information regarding DS	2.65 ± 1.049	2.96 + 0.993	2.14 + 0.929	6.827 ***
Carefully read nutritional information on DS package	2.75 ± 1.083	3.04 + 1.043	2.25 + 0.957	6.327 ***
Spend time on reading information regarding nutritional benefits of DS	2.39 ± 1.027	2.64 + 1.063	1.96 + 0.803	5.642 ***
Read or search for information regarding health or nutrition	2.36 ± 1.079	2.58 + 1.113	2.00 + 0.918	4.467 ***
**Future behavioral intention**				
I will purchase DS in the future	2.78 ± 1.081	3.15 + 0.989	2.16 + 0.916	8.311 ***

*** *p* < 0.001; ^a^ Reverse coding.

**Table 4:** Relationship between health conditions, consciousness, involvement and future behavioral intention.

Constructs	Health Conditions	Consciousness	Involvement	Future Purchase Intention
Health conditions	1	0.290 **	0.194 **	0.106
Consciousness		1	0.274 **	0.218 **
Involvement			1	0.647 **
Future purchase intention				1

** *p* < 0.01; Significances at two-tails.

**Table 5:** Multiple regression results of behavioral intentions.

Predictors	B	*SE*	*T*
Constant	0.743		2.336 *
Health conditions	−0.053	0.075	−0.704
Health consciousness	0.092	0.052	1.029
Health involvement	0.756	0.629	12.721 ***
Knowledge	−0.006	−0.004	−0.094

Note: F = 46.456 ***, R^2^ = 0.406, Adjusted R^2^ = 0.400; * *p* < 0.05, *** *p* < 0.001.
